# Hyperkalemia Presenting as Sinus Bradycardia, Junctional Rhythm and Atrial Bigeminy

**DOI:** 10.7759/cureus.6439

**Published:** 2019-12-21

**Authors:** Balaram Krishna J Hanumanthu, Yashasvi Chugh, Michael Grushko, Robert T Faillace

**Affiliations:** 1 Cardiology, Mount Sinai Beth Israel Medical Center, New York, USA; 2 Cardiology, Mount Sinai St. Luke's, New York, USA; 3 Cardiology, Albert Einstein College of Medicine/Jacobi Medical Center, Bronx, USA

**Keywords:** junctional rhythm, atrial bigeminy, hyperkalemia, bradycardia hyperkalemia

## Abstract

The spectrum of electrocardiographic changes seen with hyperkalemia is known to progress gradually with increasing serum levels of potassium. Initial changes are limited to peaked T waves and QT shortening, which subsequently progress to prolonged QRS/QT intervals, and finally sinus arrest, sinus bradycardia and asystole. We report a unique case of severe sinus bradycardia with atrial bigeminy and junctional rhythm in the setting of moderate hyperkalemia, a rarely reported electrocardiographic finding.

## Introduction

Atrioventricular (AV) junctional rhythms result from enhanced automaticity or reentry at the AV node, or during periods of sinus bradycardia when the sinus rate is slower than that of the AV node’s pacemaker cells. Moderate hyperkalemia (6.0-6.5 mEq/L) is associated with peaked T waves and prolonged QT intervals. Sinus arrest and severe sinus bradycardia usually require higher serum potassium levels (8 mEq/L) because the sinoatrial nodal cells are relatively resistant to electrolyte disturbances compared with other cardiomyocytes [1,2]. We report a case of severe sinus bradycardia with atrial bigeminy and junctional rhythm in the setting of moderate hyperkalemia.

## Case presentation

**Figure 1:**
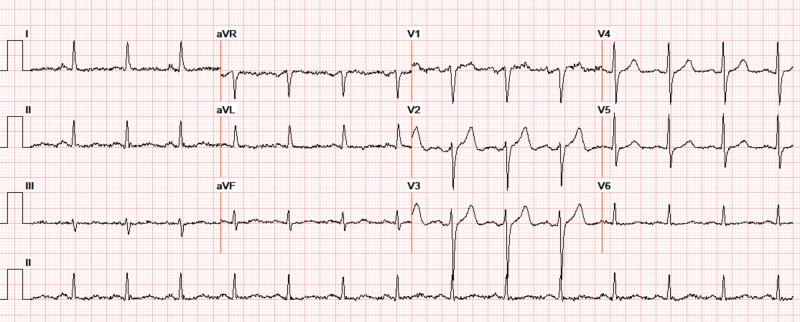
Electrocardiogram at admission, normal sinus rhythm, axis -30 and normal waves and intervals (serum potassium 4.7 mEq/L, magnesium 2.1 mEq/L, BUN 64 mg/dL, creatinine 2.7 mg/dL). BUN: blood urea nitrogen

**Figure 2:**
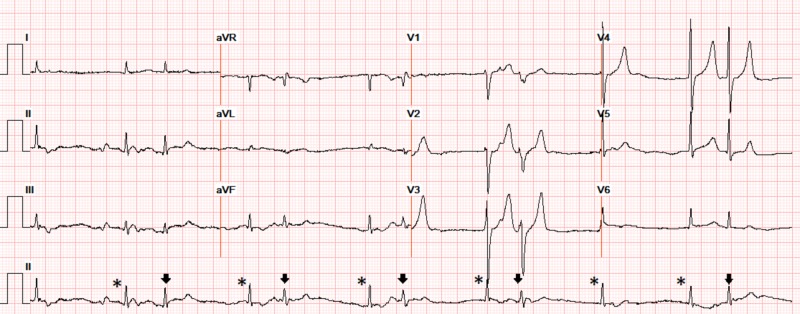
Severe sinus bradycardia with competing junctional rhythm ( * without preceding q waves) at 52 bpm, atrial bigeminy (arrow) with incomplete left bundle branch block (QRS 110 ms), and normal QTc morphology coupled regularly at intervals of 600 ms with peaked T waves in V2-4 (serum potassium 5.8 mEq/L, magnesium 1.9 mEq/L, BUN 96 mg/dL, creatinine 4.3 mg/dL). BUN: blood urea nitrogen

**Figure 3:**
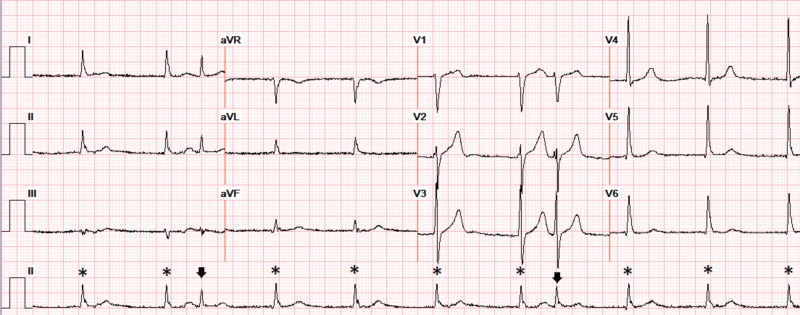
Severe sinus bradycardia with competing junctional rhythm (* without preceding p waves) (60 bpm), with atrial quadrigeminy (arrow) coupled at 550 ms with normal T waves and normal intervals (serum potassium 4.6 mEq/L, magnesium 2.0 mEq/L, BUN 61 mg/dL, creatinine 3.8 mg/dL). BUN: blood urea nitrogen

A 73-year-old gentleman with hypertension, type 2 diabetes mellitus, stage 4 chronic kidney disease, heart failure with left ventricular ejection fraction of 45% and hypothyroidism presented with progressive biventricular failure. The patient was initially in normal sinus rhythm (Figure 1). After diuretics were initiated, his renal function worsened over 72 hours with an elevation in blood urea nitrogen/creatinine (from baseline of 39/2.8 to 96/4.3 mg/dL) and potassium increased from 4.7 to 5.8 mEq/L. Other electrolytes (serum sodium (137-142 mEq/L), calcium (8-9 mg/dL) and magnesium (1.8-2.6 mg/dL)) remained within normal limits. The patient developed severe sinus bradycardia, with competing junctional rhythm at 52 bpm. Also noted were bigeminal atrial premature complexes (APCs) conducted with an incomplete left bundle branch block (QRS interval 110 ms), at regularly coupled intervals of 600 ms, along with peaked T waves (Figure 2). He was hemodynamically stable but admitted to the cardiac care unit where he was treated with hemodialysis resulting in restoration of normal sinus rhythm with resolution of APCs and normalization of T waves. However, the patient continued to have an episodic junctional rhythm (Figure 3), but at a rate of 60 bpm, with atrial quadrigeminy coupled at 550 ms with normal T waves despite normal serum potassium (4.6 mEq/L) the next day and subsequently resolved over the next few days. 

## Discussion

**Figure 4:**
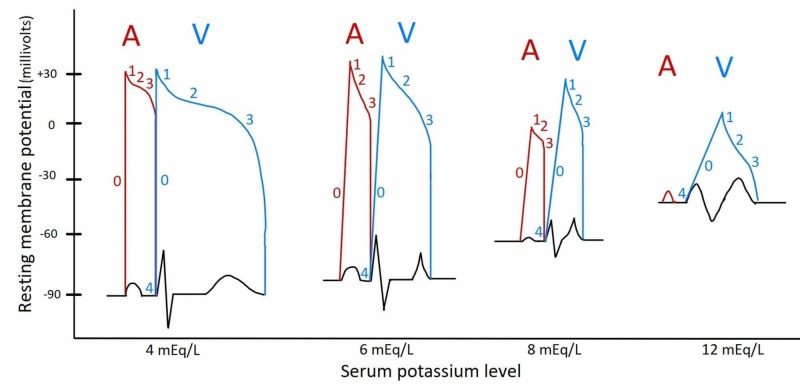
Illustration of EKG changes and superimposed atrial and ventricular action potentials at various serum potassium levels. As potassium levels increase, resting membrane potential decreases (phase 4), potassium efflux increases (phase 3 velocity) and finally phase 0 slope increases, decreasing action potential duration initially but increasing action potential at very high levels. Surface electrocardiogram shows T wave tenting and QT shortening initially but then leads to QRS widening and sinus arrest. EKG, electrocardiography

This patient's electrocardiography (EKG) reveals transient severe sinus bradycardia with junctional escape at a lower potassium level than previously reported and capture bigeminy not reported with hyperkalemia. Resting membrane potential (RMP) mainly depends on the electrochemical potential generated by the concentration gradient of potassium across the cell membrane [3]. Hyperkalemia causes the RMP to become less negative due to decreased transmembrane concentration of potassium and has two prominent effects: it brings the RMP closer to threshold and increases potassium efflux (IKr) by increasing velocity of phase 3 repolarization [3]. This initially causes shortening of action potential duration (ST-T segment depression, peaked T waves and QT interval shortening). As the RMP approaches threshold, the myocardium becomes hypoexcitable, which reduces sodium influx resulting in decrease of both the rate of rise and voltage of phase 0 of the action potential. These effects in turn reduce the speed of propagation of the action potential through the myocardium (wide QRS, prolonged PR interval) (Figure 4) [3].

Absence of p waves in this patient has two possible explanations. The first possibility being severe sinus bradycardia (or "sinus arrest") due to a hypoexcitable pacemaker with junctional escape beat conducting to the ventricle. The second explanation is direct sinoventricular conduction through interatrial fibers bypassing atrial tissue due to non-excitability of atrial myocardium (sinoatrial block). These occur at higher potassium levels (>8 mEq/L) [1] because the sinoatrial and atrioventricular nodes are relatively resistant to hyperkalemia due to their sympathetic innervation [2]. This has been reported in milder hyperkalemia (5.5 mEq/L) as well, albeit latent sinus node dysfunction was not excluded in that patient [4]. Hyponatremia and hypocalcemia have been shown to increase the sensitivity of the heart to the effects of hyperkalemia [3]. However, this patient’s serum sodium, calcium and magnesium levels were within normal limits. Junctional rhythm has been reported in the setting of hyperkalemia (6.5 mEq/L) with concurrent beta-blocker therapy, but our patient had neither been on AV node nor on beta-blocking agents [5]. Atrial bigeminy (and later quadrigeminy) in this patient is indicative of sinoatrial block allowing for a focus below the sinoatrial node to escape and allow for "escape capture bigeminy" seen in this patient. This is seen with digoxin, AV node blocking medications and sick sinus syndrome, but has not been reported due to hyperkalemia [6]. Sick sinus syndrome is not excluded especially given his age and diabetes, but temporal correlation of resolution of the EKG abnormalities with management of hyperkalemia suggests hyperkalemia to be more likely. The "milder" (faster escape and quadrigeminy as opposed to bigeminy earlier) recurrence of this rhythm briefly with a normal potassium level suggests the possibility of intracellular or pericellular hyperkalemia affecting conduction despite "normal" potassium levels. This might explain the occurrence of these conduction abnormalities in this patient at lower than usually expected as well.

## Conclusions

Although infrequent, moderate hyperkalemia can present with a rare combination of atrial bigeminy and junctional rhythm due to severe sinus bradycardia as demonstrated by the findings of our case. It is likely that although serum potassium levels were low, the relative intracellular or pericellular hyperkalemia may have affected the patient's conduction system, leading to the EKG changes. 

## References

[1] Bashour TT, Cheng TO (1975). Evidence for specialized atrioventricular conduction in hyperkalemia. J Electrocardiol.

[2] Monir G, Dreifus LS, Gursoy AS, Kutalek SP (1999). Escape capture bigeminy: a manifestation of sinoatrial conduction block. J Electrocardiol.

[3] Surawicz B (1967). Relationship between electrocardiogram and electrolytes. Am Heart J.

[4] Mehta N, Chhabra V, Khan I (2001). Sinus arrest or sinoventricular conduction in mild hyperkalemia. J Emerg Med.

[5] Isabel J, Champion JC (2006). Junctional escape rhythm secondary to acute hyperkalemic renal failure in the setting of concurrent beta-blocker therapy. JAAPA.

[6] Vassalle M, Greineder JK, Stuckey JH (1973). Role of the sympathetic nervous system in the sinus node resistance to high potassium. Circ Res.

